# The Potential of Siglecs and Sialic Acids as Biomarkers and Therapeutic Targets in Tumor Immunotherapy

**DOI:** 10.3390/cancers16020289

**Published:** 2024-01-10

**Authors:** Haokang Feng, Jiale Feng, Xu Han, Ying Ying, Wenhui Lou, Liang Liu, Lei Zhang

**Affiliations:** 1 Department of Pancreatic Surgery, Zhongshan Hospital, Fudan University, Shanghai 200032, China; hkfeng22@m.fudan.edu.cn (H.F.); 23211210049@m.fudan.edu.cn (J.F.); han.xu1@zs-hospital.sh.cn (X.H.); 17301050038@fudan.edu.cn (Y.Y.); lou.wenhui@zs-hospital.sh.cn (W.L.); 2 Cancer Center, Zhongshan Hospital, Fudan University, Shanghai 200032, China; 3 Department of General Surgery, Zhongshan Hospital, Fudan University, Shanghai 200032, China; 4 The Shanghai Geriatrics Medical Center, Zhongshan Hospital MinHang MeiLong Branch, Fudan University, Shanghai 200032, China

**Keywords:** Siglecs, immune checkpoint inhibitors, sialylation, immune cells, immune evasion

## Abstract

**Simple Summary:**

Sialic acid dysregulation is closely associated with the occurrence and development of tumors. Sialic acid-binding immunoglobulin-like lectins (Siglecs) are a class of receptors that recognize sialic acid and are widely expressed on the surface of various immune cells. In addition to exhibiting immune checkpoint inhibitory functions, Siglecs have a critical role in immune self-recognition and non-self-recognition. Some immune checkpoint inhibitors targeting specific Siglecs have entered clinical trials. However, the efficacy and potential side effects of Siglecs have not been comprehensively analyzed. Previous studies have reported the role of Siglecs in tumors, but the underlying mechanisms have not been elucidated. The elucidation of mechanisms through which Siglecs promote tumor immune escape will enable the development of novel therapeutic strategies. This review aims to describe the complex interactions between Siglecs and their sialic acid ligands in the tumor immune environment and outline the Siglec-associated pathways for developing immunotherapeutic interventions.

**Abstract:**

The dysregulation of sialic acid is closely associated with oncogenesis and tumor progression. Most tumor cells exhibit sialic acid upregulation. Sialic acid-binding immunoglobulin-like lectins (Siglecs) are receptors that recognize sialic acid and are expressed in various immune cells. The activity of Siglecs in the tumor microenvironment promotes immune escape, mirroring the mechanisms of the well-characterized PD-1/PD-L1 pathway in cancer. Cancer cells utilize sialic acid-linked glycans to evade immune surveillance. As Siglecs exhibit similar mechanisms as the established immune checkpoint inhibitors (ICIs), they are potential therapeutic targets for different forms of cancer, especially ICI-resistant malignancies. Additionally, the upregulation of sialic acid serves as a potential tumor biomarker. This review examines the feasibility of using sialic acid and Siglecs for early malignant tumor detection and discusses the potential of targeting Siglec–sialic acid interaction as a novel cancer therapeutic strategy.

## 1. Introduction

The tumor microenvironment (TME) exhibits a complex immune landscape owing to immune cell dysfunction, which promotes the progression, invasion, and metastasis of malignant tumors [1]. Immune checkpoint inhibitors (ICIs) target the impaired immune checkpoints, effectively neutralizing suppressive signals that inhibit T cell activation and amplifying the anti-tumor immune response [1]. ICIs are monoclonal antibodies that exert therapeutic effects by targeting inhibitory receptors on the T cell surface. Alternatively, ICIs can modulate Fc receptor interaction and T cell metabolism [2]. The approval of ICIs as individual or combinatorial therapies by the Food and Drug Administration has improved the treatment success in several malignancies [3]. Antibodies, such as anti-CTLA4, anti-PD-1, and anti-PD-L1 antibodies, have provided transformative, enduring clinical benefits for a subset of patients with cancer. However, this subset of patients accounts for a small proportion of cancer cases. Most patients, especially those with pancreatic ductal adenocarcinoma (PDAC), exhibit an ICI refractory phenotype [4,5]. This resistance can be attributed to both inherent tumor cell factors and extrinsic TME components. However, the comprehensive molecular mechanisms underlying ICI resistance have not been elucidated [6]. Thus, there is a need to develop alternative ICI strategies for cancer.

Sialic acids, ubiquitously expressed in human cells, play a crucial role in signal transduction on the cell surface. The regulatory pathways of sialic acids involve both epigenetic (via post-transcriptional modifications) and genetic pathways (via glycosyltransferase expression). Hypersialylation, a hallmark of various malignancies, autoimmune disorders, and infections, results from an augmented metabolic influx of sialic acids along with the upregulation of sialylation-related enzymes (sialyltransferases [STs]) and the downregulation of desialylation-related enzymes (sialidases) [7]. Recent studies have reported the role of sialylation in cancer progression. Sialic acid-binding immunoglobulin-like lectins (Siglecs), which are predominantly expressed on the white blood cell surface, are critical for the immune-mediated distinction between self and non-self antigens and exhibit immune checkpoint inhibitory activities [8]. Siglecs are reported to serve as regulators of immune surveillance with potential applications in cancer immunotherapy. Hypersialylation, when manifested on malignant cells, can enhance binding to inhibitory Siglecs on immune cells, thus perpetuating immunosuppression. This review aimed to summarize the interactions between Siglecs and their sialic acid ligands within the tumor immune microenvironment and discuss Siglec-targeting immunotherapeutic interventions.

## 2. Siglecs: Classification, Structure, Function, and Biological Regulation

**Figure 1:**
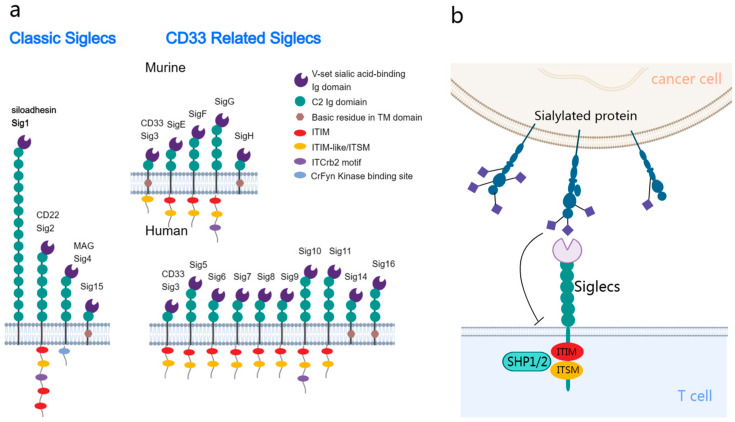
Structural and functional delineation of sialic acid-binding immunoglobulin-like lectins (Siglecs) in the tumor immune microenvironment. (**a**) Siglecs classification: the conserved subtype (**left**) is shared between humans and mice, with it exhibiting universal structural attributes. The CD33-related subtype (**right**) exhibits differential expression between humans and mice. Most Siglecs are characterized by the presence of one or more cytoplasmic immunoreceptor tyrosine-based inhibitory motif (ITIM) or ITIM-like domains. (**b**) Interaction dynamics: tumor cells present sialic acid glycans, which interact with the inhibitory Siglecs on the immune cell surface. This interaction promotes the phosphorylation and recruitment of SHP1/SHP2, activating the inhibitory signaling pathways in the associated immune cells.

Siglecs are a group of receptors expressed on the membrane of leukocytes. Additionally, Siglecs belong to the I-type lectin family of the immunoglobulin superfamily and can recognize and bind to sialic acids on glycoproteins and glycolipids. In humans, 14 distinct Siglecs have been identified, which can be classified into the following two primary subfamilies: the conserved group and the rapidly evolving CD33-related group (Figure 1a) [9].

The structure of Siglecs harbors an N-terminal V-set domain that facilitates the binding of sialic acids [10]. This domain can interact with multiple immunoglobulin ((Ig)-like domains, which are similar to the variable (V) and constant (C) domains in antibodies. The ligand binding sites of Siglecs harbor a positively charged arginine, which facilitates sialic acid binding [11]. The transmembrane domain adjacent to the V-set domain ends with a cytoplasmic tail. Siglec-1 lacks intracellular motifs, whereas other Siglecs have motifs, such as immunoreceptor tyrosine-based inhibitory motifs (ITIMs) and ITIM-like sequences [12].

Siglecs exhibit dual functions. Most Siglecs transduce inhibitory signals upon ligand binding, while some Siglecs, including Siglec-14/15/16, can transduce activation signals [13]. The phosphorylation of ITIMs, which is critical for the function of Siglecs, results in the recruitment of the phosphatases SHP1 and SHP2 [14], modulating vital signaling pathways in immune cells (Figure 1b) [15]. These phosphatases inhibit the activation of several signaling molecules, promoting various biological behaviors depending on the Siglec type and host cell [16,17,18,19]. Similar to PD1-mediated immune inhibition, this mechanism potentiates the potential therapeutic effects of Siglecs on tumors [20]. Compared to the interaction between PD-1 and PD-L1, sialoglycan ligands may bind to Siglec receptors on the same cell (cis) rather than just on other cells (trans) [21].

The regulation of Siglecs depends on their affinity for different sialylated glycans, effectively modulating immune cell functions. Immunoreceptor tyrosine-based activation motif-bearing Siglecs can tether the DAP12 protein, catalyzing the recruitment of SYK and activating the cell proliferation pathways [22,23].

Thus, Siglecs are intricate structural and functional entities in the immune landscape. After the interaction of inhibitory Siglec receptors with sialoglycan ligands, Siglec receptors undergo phosphorylation, and SHP1/2 phosphatases are recruited, thereby inhibiting intracellular immune signaling. In comparison, this is mechanistically similar to the interaction of PD-1 with PD-L1, where PD-1 can also recruit SHP1/2 phosphatases to inhibit intracellular immune signaling [24]. Thus, the elucidation of the mechanisms of Siglecs can enable their application in cancer immunotherapy.

## 3. Sialylation Dysregulation and Siglec-Mediated Immune Evasion in Tumorigenesis

During tumorigenesis, heightened expression of Siglecs on immune cell surfaces, especially post CD3 and CD28 activation in Treg cells, has been identified [25,26]. The underlying mechanisms and associated cytokines in the tumor microenvironment (TME) that facilitate immune evasion remain to be elucidated [27]. Aberrant sialoglycan expression, resulting from dysregulated glycan synthesis, encompasses two primary forms: the emergence of sialic-acid modified truncated O-glycans revealing the tumor-associated carbohydrate antigen sTn [28], linked to sialyltransferase ST6GalNAc1 overexpression and COSMC gene alterations [29,30], and augmented sialic acid residues on cancer cell surfaces, attributed to modulated sialyltransferase and neuraminidase activities [31]. This augmented sialylation has implications for immune evasion [32], positioning Siglecs as pivotal glycan immune checkpoints in the TME.

During tumorigenesis, the expression of Siglecs is upregulated on the immune cell surfaces, especially post-CD3 and CD28 activation in T regulatory cells [25,26]. The mechanisms and associated cytokines in the TME that facilitate immune evasion have not been elucidated [27]. Sialoglycan dysregulation resulting from disrupted glycan synthesis can be attributed to the following two primary pathways: the emergence of sialic acid-modified truncated O-glycans, which reveals the tumor-associated carbohydrate antigen sTn [28] and is linked to ST6GALNAC1 upregulation and C1GALT1C1 alterations [29,30], and augmented sialic acid residues on the cancer cell surfaces, which is attributed to aberrant sialyltransferase and neuraminidase activities [31]. The upregulation of sialylation has implications for immune evasion [32]. Thus, Siglecs are pivotal glycan immune checkpoints in the TME.

### 3.1. Siglecs and Ligand-Mediated Immune Evasion Mechanisms in Oncogenesis

Tumor progression is characterized by glycosylation shifts. Aberrations in sialic acid expression or sialoglycan structure in cancer cells disrupt Siglec-sialic acid signaling. Siglecs are critical for modulating the anti-tumor immune landscape of the TME [27,33,34]. After interaction with sialic acid, inhibitory Siglecs undergo SRC kinase-mediated ITIM tyrosine phosphorylation, promoting SHP1/SHP2 recruitment (similar to the PD-1/PD-L1 pathway) and, subsequently, inhibition pathways, such as the SPRY pathway. This inhibition activates the Ras/Raf/MAPK oncogenic cascade. The NK-κB pathway [35], which is activated by PTPN11 and inhibited by PTPN6, modulates cytokine levels via the JAK-STAT pathway [36,37]. Such regulations are potentially negated by Src Kinase Associated Phosphoprotein 2 (SKAP2), implying a role in inflammation-driven oncogenesis [38]. Within immune cell modulation, SHP2 and SHP1 respectively influence M1 phenotype activation in TAM and T-cell inactivation [39,40].

### 3.2. Siglecs in Immunosuppression across Cell Types

Siglecs, which are widely and differentially expressed on the immune cell surface, are markers for differentiating immune subsets. Exposure to some factors in the TME stimulates the expression of Siglecs on the immune cell surface [34,41]. This dysregulated expression is further associated with the occurrence and development of tumors, emphasizing the importance of understanding Siglec-mediated cellular mechanisms in cancer immunity. Table 1 delineates the presence of various Siglecs on immune cells along with their as-sociated functions.

#### 3.2.1. Siglecs and NK Cells

Predominantly, human NK cells express Siglec-7 and -9, with TME-associated NK cells showing elevated expression of Siglec-9, particularly in the CD56dim CD16 + subset [9,42]. These CD56dim NK cells, constituting approximately 90% of NK cells in the peripheral blood and spleen, are distinguished by their cytotoxin expression: perforin and granzymes [43,44]. While Siglec-7 is an acknowledged NK cell marker [45], Siglec-9 marks a specific CD56dim NK subtype [1]. Siglec-7 and -9 ligands, pervasive on tumor cells, confer protection against NK cell-mediated attacks for tumor cells. Engaging these Siglecs diminishes NK activity [25,42] and tumor–NK encounters, inducing Siglec-7 ligand synthesis. Monoclonal antibodies that target the glyco-immune interactions of Siglec-7 or biologics that engage NK cells have been shown to elicit robust antitumor immune responses in ovarian cancer [46]. As an obvious example, Siglec-9 interaction with ovarian cancer cell surface mucin MUC16 potentially suppresses anti-tumor responses [47]. The subsequent formation of the immunological synapse delays the endocytosis of cell surface glycoconjugates and facilitates Siglec-7 ligand accumulation, activating inhibitory signaling [48]. Blocking Siglec-7 and -9 augments NK activity [49]. In multiple myeloma, SELPLG serves as a Siglec-7 ligand, enabling immune evasion [50]. This suggests that SELPLG is a vital target for developing NK cell therapies. Additionally, the interaction of Siglec-7 on the NK cell surface with group B streptococci β-protein inhibits pyroptotic cell death [51].

#### 3.2.2. Siglecs and B Cells

B cells predominantly express CD22 (also called Siglec-2), which is a conserved receptor that regulates B cell differentiation, signaling, and functions, such as immune tolerance [8,52]. Mouse Siglec-G is similar to human CD22. Human Siglec-10 exhibits significant sequence homology with Siglec-G and modulates B cell receptor signaling, suppressing the Ca^2+^ response [53,54]. In autoimmune rheumatoid arthritis, CD22 modulates B cell immunity [55]. The preferred ligand of CD22 (α2,6 sialylated glycan) is synthesized by ST6GAL1, promoting B cell immune suppression. The anti-CD22 monoclonal antibody SM03 mitigates the suppression of NF-κB signaling induced by CD22 and SHP1, restoring B cell responsiveness in malignancies [56]. Chimeric antigen receptor T cell (CAR-T) therapies predominantly target B cell CD22, suppressing the growth of acute lymphoblastic leukemia [57].

#### 3.2.3. Siglecs and T Cells

Under basal conditions, the expression of Siglec in T cells is lower than that in B cells. However, the expression levels of Siglec-7 and Siglec-9 are markedly upregulated in some subsets [34,58]. Siglec upregulation in T cells is largely driven by pathological conditions [59]. Although Siglec diversity on the T cell surface is higher than that on the B cell surface, Siglec-7 functions as a glyco-immune checkpoint in memory CD8+ T cells during oncogenesis and in T cell disorders [60]. Some studies have reported that Siglec-9 potentiates T cell activity in the TME [58], whereas other studies have reported the inhibitory effect of Siglecs-9 on T cell activity [61]. Siglec-5 and Siglec-14 are abundantly expressed on the CD8+ T cell surface post-anti-CD3 activation [58]. In tumors, sialylated IgG suppresses T cells by interacting with Siglec-7 [62]. The CD52–Siglec-10 interaction suppresses CD4+ T cell immunity [63]. Siglec-15, which is involved in osteoclastogenesis and immune regulation, is upregulated in various cancers [33,35]. The deletion of Siglec-15 enhances tumor-specific T cell responses [64]. T cell-engaging bispecific antibodies (T-BiAbs), such as JML-1 and RC-1, target Siglec-6 on the T cell surface, exerting therapeutic effects on CLL [65]. Mouse studies have reported the upregulation of sporadic Siglec-G expression and pathology-driven Siglec-F [66,67], especially in tumors [68].

#### 3.2.4. Siglecs and Macrophages

Macrophages exhibit tissue-specific Siglec profiles during differentiation. Monocytes inherently express several Siglecs. Siglec-1, which is modulated by the cytokines IFN-α and IFN-γ [69], and Siglec-11 expression is localized to immune-rich sites and specific organs [70,71]. Additionally, CD33 (also called Siglec-3) and Siglec-16 are upregulated in some healthy and cancerous tissues [72,73]. Siglec-15 is a potential ICI target owing to its upregulation in TAMs [74,75].

PDAC cells, which exhibit upregulated levels of ST3GAL1 and ST3GAL4, promote monocyte differentiation via Siglec-mediated signaling [76,77], which is also observed in lung squamous cell carcinoma [78]. Importantly, it is noteworthy that Siglec-9 plays a crucial role in monocyte differentiation into macrophages, and Siglec-9-mediated interactions in macrophages can further suppress anti-tumor T cell responses [79]. Barkal et al. reported that the CD24/Siglec-10 axis is a macrophage phagocytosis checkpoint [80,81]. The authors postulated that the upregulation of CD24 in cancers, such as ovarian cancer and triple-negative breast cancer, facilitates immune escape. The inhibition of the CD24/Siglec-10 axis markedly promoted phagocytosis [74]. In murine models, Siglec-E is predominantly expressed under physiological and tumorigenesis conditions. The knockout of Siglec-E suppresses tumor growth and modulates macrophage polarization [76].

#### 3.2.5. Siglecs and Dendritic Cells (DCs)

Dendritic cells (DCs), functioning as specialized antigen-presenting cells, play a pivotal role in instigating the adaptive immune response and are integral to immuno-therapeutic interventions [82]. Recent investigations have shed light on the involvement of Siglecs receptors in discrete DC subpopulations. Particularly noteworthy is the inhibitory role of Siglec-G on murine DCs, which has been discerned as impeding DCs’ cross-presentation by exerting a discernible impact on the formation of the cross-presenting MHC I-peptide complex [83]. Furthermore, heightened expression of Siglec-E in murine DCs has been associated with compromised DC maturation [84]. Significantly, Siglec-7, Siglec-9, and Siglec-10 manifest elevated expression levels on classical conventional DCs (cDCs) in the context of cancer patients [77]. In the domain of lung cancer, the expression of Siglec-15 has been observed to exhibit a substantial correlation with the short-term survival rate of patients, concurrently with diminished infiltration in the immunohistochemical detection of dendritic cells [85]. Notably, Siglec-15 emerges as a potential prognostic marker. Additionally, Siglecs are intricately linked to the infective invasion of viruses. Siglec-1 mediates the infection of CCR5- and CXCR4-tropic strains to DCs [86]. Especially in the case of HIV infection, DC subsets expressing Siglec-1 have been implicated in promoting HIV-1 replication and facilitating the systemic dissemination of the virus [87]. In summation, a nuanced comprehension of the impact of inhibitory Siglecs on DCs holds promise for advancing immunotherapeutic strategies across diverse pathological contexts, including cancer and other diseases.

## 4. Cancer Therapeutic Strategies Targeting the Sialoglycan–Siglec Immune Checkpoint

**Figure 2:**
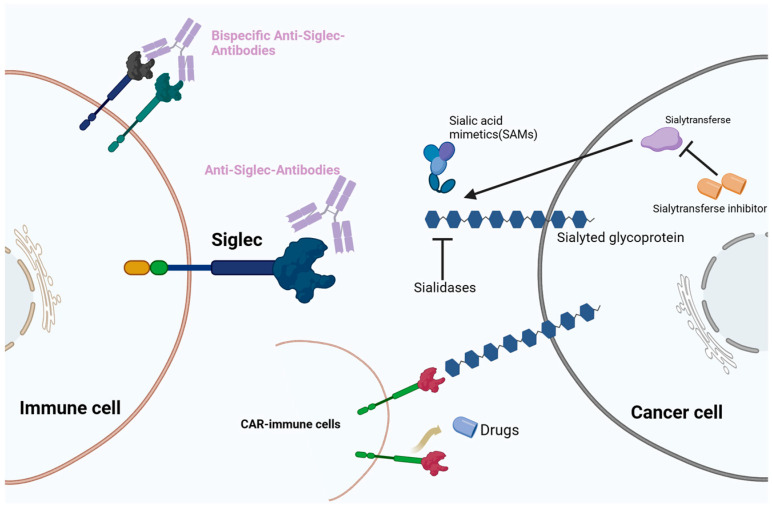
Therapeutic strategies targeting the sialic acid–sialic acid-binding immunoglobulin-like lectin (Siglec) axis. Immunotherapies include monoclonal antibodies, bispecific antibodies, chimeric antigen T cell (CAR-T) therapy, and sialic acid mimetics (SAMs).

Recent clinical trials have explored therapies targeting the Siglec glycobiome (Figure 2). The development of Siglec datasets in oncological, infectious, and autoimmune diseases has attracted attention to sialylation and its interplay with Siglecs in the field of glycobiology and novel ICI development. Under pathophysiological conditions, the sialic–Siglec axis is a promising therapeutic target for cancer.

Siglec-5, an immunosuppressor in M2-type TAMs, plays a critical role in the pathogenesis of various cancers, indicating its potential as an ICI target. Recent histological studies have revealed the mutual exclusivity between Siglec-15 and PD-L1 in specific cancers [88]. Consequently, ICIs targeting Siglec-15 may complement treatment in patients with suboptimal PD-1/PD-L1 expression or patients who are unresponsive to anti-PD-1/PD-L1 therapy. Table 2 enumerates several emergent therapies targeting Siglecs with potential clinical applications.

### 4.1. Monoclonal Antibodies Targeting Siglecs

ICIs are monoclonal antibodies (mAbs) that can target Siglecs. These mAbs, which are characterized by specificity and antibody-dependent cellular cytotoxicity, exhibit dual roles. mAbs modulate the function of immune cells expressing Siglecs and consequently improve the TME immune response. Additionally, mAbs disrupt the interaction of sialylated ligand-expressing cancer cells with Siglecs, preventing immune evasion. Clinical investigations have identified that Siglec-7, Siglec-9, Siglec-10, and Siglec-15 are promising targets, which is corroborated by in vivo and in vitro studies [42,103].

Antibodies against Siglecs can be categorized into two primary types:

Antagonistic antibodies: These antibodies bind to Siglecs, inhibiting the binding of sialylated glycans. Antagonistic antibodies disrupt the interaction between the Siglec and its ligand and prevent cancer cells from evading immune surveillance. Recent studies have demonstrated the significance of Siglec-7 and Siglec-9 in the immune evasion process, especially in the context of TAMs and NK cells [104]. Moreover, Siglec-7 and Siglec-9 are predominantly expressed in neutrophils in cancers [105]. Targeting these Siglecs is a promising strategy, and clinical validations of these targeting strategies are ongoing [106]. Siglec-10, which interacts with sialylated CD24 in some cancers, triggers a potent immune suppression pathway in TAMs. Targeting Siglec-10 or CD24 is a feasible ICI strategy [107]. Furthermore, Siglec-15, which is abundantly expressed on the TAM surface, can potentially serve as an ICI target owing to its role in the immune evasion of various cancers [108,109]. NC318, an anti-Siglec-15 mAb, has demonstrated therapeutic potential in preclinical studies and is currently undergoing phase I/II clinical evaluations [110].

Agonistic antibodies: These antibodies interact with Siglecs via cross-linking. Agonistic antibodies interact with Siglecs harboring the ITIM, especially at the non-sialylated glycan sites, activating intracellular inhibitory signal transduction [106]. The application of agonistic antibodies is suitable for autoimmune and inflammatory conditions. For example, cross-linking with Siglec-7 on the mast leukemic cell surface suppresses KIT signaling, promoting apoptosis in mast cells and mitigating inflammation [111].

### 4.2. BsAbs

Compared with mAbs, BsAbs, which can bind two discrete antigens, exhibit enhanced therapeutic efficacy and decreased adverse events. The efficacy of BsAbs has been reported in hematological malignancies [112,113]. In particular, bispecific T cell engagers specifically target leukemia cells and modulate their microenvironment [114]. Siglec-2 (CD22) is upregulated in approximately 90% of acute myeloid leukemia (AML) cases. Additionally, CD47 is commonly upregulated in AML. Based on these observations, Boyd-Kirkup et al. developed a BsAb, HMBD004, targeting CD47 + CD33+ cells [115]. This construct disrupts CD47/SIRPα signaling and macrophage phagocytosis, exerting anti-tumor effects in murine AML models. The performance of HMBD004 is higher than that of CD47 monotherapy in terms of specificity, efficacy, and safety. Furthermore, Siglec6-targeting T cell-recruiting biAb (T-biAb), which has been developed for chronic lymphocytic leukemia (CLL) [81], efficiently eliminates Siglec-6^+^ leukemia and B cells and preserves Siglec-6^−^ healthy B cells.

### 4.3. Antibody-Based Cell Depletion

Upon binding with sialylated glycans or antibodies, Siglecs undergo endocytosis, leading to the cellular internalization of the bound entities. Based on this property, cytotoxic antibodies can directly target immune cells overexpressing Siglecs. This offers a potential therapeutic avenue for hematological malignancies or modulating autoimmune disease-driven inflammatory cells. CD22 and CD33, which are consistently expressed on leukemia and lymphoma cell surfaces, are potent antibody targets in hematological conditions [116,117]. For example, inotuzumab ozogamicin, an anti- Siglec-2 conjugate combined with the toxin calicheamicin, exerts therapeutic effects on refractory B cell acute lymphoblastic leukemia and is associated with decreased cytotoxic effects [118].

Siglec-8, which is predominantly expressed on the eosinophil, basophil, and mast cell surfaces, is a target for allergic conditions [119]. However, O’Sullivan et al. have reported that Siglec-8 has targeting potential in chronic eosinophilic and mast cell leukemias [119]. The authors elucidated the endocytic pathways of Siglec-8 and demonstrated that the immunotoxin effectively targeted eosinophils and malignant mast cells in vitro, consistently inducing cell death.

### 4.4. CAR-T Therapeutic Approaches

CAR-T cells, which are genetically engineered to express chimeric antigen receptors, present a novel therapeutic avenue for targeting Siglecs. The efficacy of CAR-T therapy has been reported, especially in hematological malignancies. CAR-T cells equipped with anti- Siglec-2 (CD22) and anti- Siglec-3 (CD33) receptors exert potent therapeutic effects on leukemia and lymphoma, respectively [120,121]. Recent advances indicate that the monotherapeutic efficacy of anti-CD19 CAR-T cells can be enhanced to mitigate relapse rates when combined with bispecific CAR-T targeting CD22 and CD19 in refractory or relapsed B cell hematological malignancies [122]. CD22+ CAR-T therapy achieved an 80% complete remission rate, equitably targeting both CD19+ and CD19− B cells [123,124]. Preclinical studies have also reported the potential of Siglec-6 CAR-T therapy in inducing complete remission in the AML xenograft models using immunodeficient mice [98] as well as in exerting growth-inhibitory effects against CLL [125].

### 4.5. Sialic Acid Mimetics (SAMs) in Siglec Targeting

SAMs, which are synthetic molecules structurally mimicking sialylated glycoproteins, serve as high-affinity Siglec ligands. The functions of SAMs are multifaceted. SAMs can block tumor cells expressing specific Siglecs or competitively inhibit native Siglec binding. Consequently, SAMs can impair Siglec–ligand interactions, modulating Siglec signaling and immunological responses in diseases [126]. In allergic reactions, SAMs can disrupt the interaction of CD33 on the mast cell surface with allergens, inhibiting IgE release [127]. SAMs can modulate cancer immune suppression and serve as agonistic antibodies activating Siglec signaling. Additionally, SAMs have potential applications in drug delivery. For example, nanoparticles containing CD22 SAMs have been designed to deliver cytotoxic agents, such as doxorubicin to malignant B cells expressing CD22 [128]. SAMs can inhibit sialic acid expression. The fluorinated sialic acid mimetic Ac53FaxNeu5Ac disrupts sialic acid expression in tumor cells [129]. Mechanistically, Ac53FaxNeu5Ac inhibits STs, suppressing sialic acid attachment to glycans and modulating cellular sialic acid content. Empirical data from melanoma and neuroblastoma models indicate that Ac53FaxNeu5Ac decreases specific immune regulatory cells and potentiates CD8+ T cell cytotoxicity [130,131].

## 5. Siglecs as Disease Biomarkers

In addition to their role in modulating immune responses, Siglecs and their ligands are potential predictive biomarkers for diverse pathologies, including cancer, autoimmune disorders, and viral infections. In particular, CD33 (Siglec-3) is a definitive marker for myeloid leukemias [132]. In addition to their role in modulating immune responses, Siglecs and their ligands are potential predictive biomarkers for diverse pathologies, including cancer, autoimmune disorders, and viral infections. In particular, CD33 (Siglec-3) is a definitive marker for myeloid leukemias [132], and it has emerged as a vital target in leukemia therapeutics, with its expression levels prognosticating the efficacy of gemtuzumab ozogamicin (GO) in AML treatments. Elevated CD33 expression correlates with a heightened response to GO, denoting its utility as a predictive biomarker for treatment optimization in AML [133]. CD22 (Siglec-2), which is pivotal for B cell activation, serves as a therapeutic target and a reliable biomarker for B cell-derived non-Hodgkin’s lymphoma and some autoimmune disorders [134], and patients exhibiting elevated CD22 expression derive enhanced survival benefits from CD22-targeted therapeutics [135]. A focused retrospective clinical study highlighted a robust correlation between plasma Siglec-5 levels and early-stage fulminant myocarditis, indicating the biomarker potential of Siglec-5 [125]. The upregulation of soluble Siglec-5 levels in the saliva of patients with primary Sjögren’s syndrome further demonstrated the diagnostic potential of Siglec-5 [125]. CD169 (Siglec-1) expression on the monocyte surface is a diagnostic biomarker for connective tissue diseases although it is not exclusive to systemic sclerosis [136]. Additionally, Siglec-1 may have potential applications in the diagnosis of viral infections, such as severe acute respiratory syndrome coronavirus 2, respiratory syncytial virus, and HIV infections [137]. A recent study shows that Siglec-7 and Siglec-9 modulate the immune environment in pancreatic cancer via sialic acid interactions, driving macrophage differentiation and skewing their activity towards a suppressive phenotype, marked by increased PD-L1 and IL-10 expression. This axis presents a potential marker for poor prognosis in pancreatic ductal adenocarcinoma [77].

A retrospective examination of patients with esophageal squamous cell carcinoma treated with neoadjuvant chemoradiotherapy (CRT) indicated that Siglec-15 can predict the benefits of the combination of immunotherapy and CRT [138]. TCGA data analysis indicates that increased Siglec-15 expression is associated with extended overall survival in bladder urothelial carcinoma (BLCA), breast invasive carcinoma (BRCA), head and neck squamous cell carcinoma (HNSC), thyroid carcinoma (THCA), and uterine corpus endometrial carcinoma (UCEC). It also correlates with prolonged recurrence-free survival in breast invasive carcinoma (BRCA), liver hepatocellular carcinoma (LIHC), ovarian serous cystadenocarcinoma (OV), and uterine corpus endometrial carcinoma (UCEC), highlighting its significance as a prognostic biomarker and a prospective target for immunotherapy specific to these cancers [85].

## 6. Discussion and Future Perspectives

The rapid evolution of glycomics has revealed the intricate interactions between sialic acid-modified glycans and their Siglecs, contributing to studies on their oncogenic roles. Siglec is a class of receptors with distinct binding preferences, showing specificity for various sialic acid glycan chains present on the surface of mammalian cells. As sialic acid is ubiquitous on all cells, Siglecs assist immune cells in distinguishing between self and non-self. The capacity of Siglecs to guide leukocytes in distinguishing between “self” and “non-self” directly influences immune responses, tissue micro-environments, and the tumor microenvironment (TME). The ability of Siglecs to guide leukocytes in distinguishing between “self” and “non-self” directly affects immune responses, tissue microenvironments, and the TME. These findings have expanded the therapeutic horizons in oncology. However, the precise mechanisms of Siglecs must be elucidated. Preliminary strategies have targeted Siglec–sialoglycan interactions and developed ICIs that counteract immune evasion. Several ICIs targeting specific Siglecs, especially Siglec-15, have transitioned to clinical trials. A comprehensive understanding of the efficacy, potential side effects, and combinatory potential with standard therapies of Siglecs is lacking. Future studies should focus on the role of Siglecs beyond immunology and target processes, such as hypoxia and glycolysis. A holistic understanding of Siglecs will facilitate the design of precise therapeutic interventions. In addition to elucidating the immunosuppressive function of the Siglec family, the comprehensive delineation of their roles in various cell types remains a crucial research frontier. The elucidation of the mechanisms underlying Siglec-mediated tumor immune evasion is critical for future therapeutic advancements, ensuring enhanced treatment efficacy and safety.

## 7. Conclusions

The unique role of Siglec molecules in immune regulation has propelled immunology research into an innovative direction. Over the past decade, exhaustive exploration of Siglecs has not only unveiled their multifaceted functions in immune cell dynamics but has also underscored their potential as pivotal immune checkpoints. This comprehensive review intricately delves into the regulatory roles of Siglecs across various immune processes, highlighting their promising applications in conditions such as cancer, autoimmune disorders, and infections. As an exemplary member of the Siglec family, CD22 illuminates the diversity and intricacy of Siglec functions. We have conducted an in-depth study on the context-dependency of CD22 in B cell activation, comprehensively exploring its immunomodulatory functions as well as its clinical applications as a drug target [139]. Alongside CD22, we extend our focus to CD33, another prominent Siglec member, which has been scrutinized for its pivotal role in hematological malignancies, especially leukemia. CD33’s expression on myeloid progenitor cells and leukemia blasts makes it a prime target for antibody-based therapies [140], such as antibody–drug conjugates, that seek to selectively eliminate leukemic cells while sparing normal hematopoietic stem cells. This targeted approach aims to leverage CD33’s biological function to advance leukemia treatment, highlighting the critical impact of Siglecs in the development of novel cancer therapies [141]. Notably in the realm of cancer therapy, the role of Siglecs has sparked widespread interest, such as Siglec-15. Apart from NC318, which is currently undergoing clinical trials, the utilization of nanotechnology for the targeted inhibition of Siglec-15 has been identified as a significant recent research initiative. There is a high level of anticipation within the scientific community for the data that is beginning to emerge, as well as for the potential therapeutic applications that this novel strategy may yield [142,143]. We explore the expression profiles of specific Siglecs in various cancer types and their interplay with immune checkpoints like PD-L1. These insights offer valuable perspectives for pioneering novel cancer immunotherapies, particularly for patients resistant to PD-1/PD-L1 treatments [61].

Despite the tremendous potential of Siglecs in immune modulation and disease treatment, translating this concept into practical therapeutic approaches encounters a myriad of challenges. Primarily, reliable methods are imperative to detect the expression and activity of Siglecs, augmenting our comprehension of their roles in disease progression [144]. Additionally, in the development of therapeutic drugs targeting Siglecs, scrupulous consideration of selecting appropriate targets and strategies is crucial to ensure their efficacy and safety in clinical trials. Although the field of Siglec research still grapples with unsolved mysteries and technical challenges, substantial progress has been made in recent years. The immunoregulatory roles of Siglecs and their pivotal positions in diseases have positioned them as a focal point in both immunological and clinical research. Looking ahead, in-depth studies into the molecular mechanisms of Siglecs, coupled with the development of novel detection tools and treatment strategies, hold the promise of unlocking new avenues for cancer immunotherapy and the treatment of other immune-related diseases—ushering in a potential paradigm shift in the field.

## Figures and Tables

**Table 1:** Overview of Siglecs in immune cell types.

Cell Type	Predominant Siglecs	Associated Functions
NK Cells	Siglec-7, Siglec-9	Inhibition of NK cell activity; ligands confer protection
B Cells	Siglec-2 (CD22), Siglec-G, Siglec-10	Modulation of B cell differentiation; immune tolerance
T Cells	Siglec-7, Siglec-9, Siglec-5, Siglec-14	Regulation of T cell activity; diversity in pathology-driven expression
Monocytes/Macrophages	Siglec-E, Siglec-1, Siglec-10, Siglec-9, Siglec-11, Siglec-3, Siglec-16, Siglec-15,	Tissue-specific profiles; involvement in T cell differentiation
Dendritic Cells	Siglec-E, Siglec-G, Siglec-7, Siglec-9, Siglec-10, Siglec-15	Impact on DC cross-presentation; association with DC maturation

**Table 2:** Therapeutic agents targeting Siglecs.

Human	Cell Type	Diseases	Medicine
Siglec-1 (CD169/Sialoadhesion)	Macrophages	Cancer SLE Infectious diseases	
Siglec-2 (CD22)	B cells	B-cell lymphoma Type 1 diabetes Sepsis RA SLE	Epratuzumab (mAb, agonistic antibody) [89] Inotuzumab ozogamicin (Antibody-based cell depletion) [90] DT2219 (BsAb) [91] CD22-CAR-T (CAR-T) [92]
Siglec-4 (MAG)	Myelin-producing cells (Schwann cells and oligodendrocytes)	Stroke Schizophrenia Neuropathy	
Silec-15 (CD33L)	Macrophages Dendritic cells	Cancer Osteoporosis	NC318 (mAb, antagonistic antibody) [93]
Siglec-3 (CD33)	Myeloid cells	ML Myelodysplastic syndromes Alzheimer’s disease Mast cell-dependent anaphylaxis Systemic mastocytosis	Lintuzumab (mAb, antagonistic antibody) [94] Gentuzumab ozogamicin (antibody-based cell depletion) [95] AMG330 (BsAb) [96] CD33 CAR-T (CAR-T) [97]
Siglec-5 (CD170)	Monocytes Neutrophils B cells Tumor-infiltrating T c	Cancer Neutrophil disorders	
Siglec-6 (CD327)	Mast cells B cells Dendritic cells	Allergy Trophoblastic diseases Preeclampsia	Siglec 6 CAR-T (CAR-T) [98]
Siglec-7 (CD328)	NK cells Monocytes Mast cells Basophils Neutrophils T cells	Alzheimer’s disease Systemic mastocytosis Mast cell leukemia Cancers	Ganglioside GD3 (BsAb) [99] Siglec 7 CAR-T (CAR-T) [100]
Siglec-8	Eosinophils Mast cells Basophils	Allergic asthma Eosinophilic gastric Chronic urticaria	
Siglec-9 (CD329)	Macrophages NK cells Monocytes Dendritic cells Neutrophils T cells	Asthma COPD RA Cancer	Gatipotuzumab (mAb, antagonistic antibody) [101] Siglec 9 CAR-T (CAR-T) [100]
Siglec-10 (SLG2)	B cells NK cells Monocytes Dendritic cells CD4+ T cells	Sepsis Cancer	Alemtuzumab (mAb, antagonistic antibody) [102]
Siglec-11	Macrophages Microglia B cells	Alzheimer’s disease Acute tissue injury	
Siglec-12	Macrophages	Cancer	
Siglec-14	Monocytes Neutrophils	SLE COPD GBS infection	
Siglec-15	Microglia Macrophages	Schizophrenia	
